# Design of Beta-2 Microglobulin Adsorbent Protein Nanoparticles

**DOI:** 10.3390/biom13071122

**Published:** 2023-07-14

**Authors:** Justin E. Miller, Roger Castells-Graells, Mark A. Arbing, Aldo Munoz, Yi-Xiao Jiang, Charlize T. Espinoza, Brian Nguyen, Paul Moroz, Todd O. Yeates

**Affiliations:** 1 Molecular Biology Institute, University of California, Los Angeles, CA 90095, USA; 2 UCLA-DOE Institute for Genomics and Proteomics, Los Angeles, CA 90095, USA; 3 Department of Chemistry and Biochemistry, University of California, Los Angeles, CA 90095, USA; 4 School of Medicine, Curtin University, Perth, WA 6845, Australia

**Keywords:** protein design, beta-2 microglobulin (B2M), amyloidosis, dialysis, middle molecules, protein cages, nanoparticles

## Abstract

Beta-2 microglobulin (B2M) is an immune system protein that is found on the surface of all nucleated human cells. B2M is naturally shed from cell surfaces into the plasma, followed by renal excretion. In patients with impaired renal function, B2M will accumulate in organs and tissues leading to significantly reduced life expectancy and quality of life. While current hemodialysis methods have been successful in managing electrolyte as well as small and large molecule disturbances arising in chronic renal failure, they have shown only modest success in managing plasma levels of B2M and similar sized proteins, while sparing important proteins such as albumin. We describe a systematic protein design effort aimed at adding the ability to selectively remove specific, undesired waste proteins such as B2M from the plasma of chronic renal failure patients. A novel nanoparticle built using a tetrahedral protein assembly as a scaffold that presents 12 copies of a B2M-binding nanobody is described. The designed nanoparticle binds specifically to B2M through protein–protein interactions with nanomolar binding affinity (~4.2 nM). Notably, binding to the nanoparticle increases the effective size of B2M by over 50-fold, offering a potential selective avenue for separation based on size. We present data to support the potential utility of such a nanoparticle for removing B2M from plasma by either size-based filtration or by polyvalent binding to a stationary matrix under blood flow conditions. Such applications could address current shortcomings in the management of problematic mid-sized proteins in chronic renal failure patients.

## 1. Introduction

For patients afflicted by chronic renal failure (CRF), hemodialysis is a lifesaving and life-enhancing therapy wherein a hemodialyzer aims to fulfil the blood-filtering function of healthy kidneys. However, the methods and technologies used to filter some waste products from the plasma of CRF patients are poorly effective, resulting in high levels of many uremic toxins that can ultimately diminish long-term prognosis [1]. This limitation in current technology is in large part due to the lack of selectivity with which some waste products are removed from patient dialysate; discrimination of waste proteins from required beneficial proteins occurs primarily by size, diffusion across a semipermeable membrane or via membrane adsorption. Established dialysis technologies are most effective at removing small uremic toxins and balancing volume and electrolytes but can struggle to remove larger molecules, especially middle-sized proteins (2 to 4 nm)—which diffuse more slowly—while retaining necessary serum components such as albumin and some immunoglobulins. As a consequence, patients on long-term dialysis therapy often experience an unintended increase in the concentration of proteins and molecules that fall in between the molecular weight of small molecules and larger proteins, often referred to as ‘middle molecules’.

One of these difficult-to-remove middle molecules is the 11.8 kDa protein beta-2 microglobulin (B2M). The presence of many other middle molecules such as—albumin, immunoglobulins, transthyretin, tumor necrosis factor, various interleukins and other cytokines—all of similar size, some of which must be retained while others must be removed, sets a difficult task for size-based filtration systems that do not recognize specific targets.

B2M is a subunit of the major histocompatibility complex I (MHC-I), which resides on the surface of cell membranes of all nucleated cells, and also plays a role in normal immunologic function. B2M is a member of a broad class of proteins known to transition from a functional folded state to a misfolded amyloid state [2,3,4,5]. Approximately 2–4 mg/kg/day [6] of B2M is produced per day with 99% [7] excreted by the healthy kidney so that the normal plasma concentration of B2M is less than 2 µg/mL. CRF leads to reduced renal excretion of B2M resulting in chronically elevated plasma concentrations of 50–100 µg/mL, in which the amyloid state will commonly arise. In CRF, the high concentration of circulating B2M often results in amyloid deposition in a condition known as dialysis-related amyloidosis (DRA). DRA is characterized by the formation of insoluble deposits of B2M throughout the body, resulting in pain and physiological dysfunction, with effective treatments mostly limited to pain management or surgical intervention [8]. Because of the limited treatment options for DRA, the development of methods to control blood B2M concentrations in patients on hemodialysis is of considerable medical importance.

Many of the efforts to improve the removal of middle molecules such as B2M during hemodialysis rely on tuning the characteristics of the membranes used or on using non-specific protein-adsorbent media. These innovations have included changing the pore size or chemistry of hemodialyzer membranes, or introducing a hydrophobic protein-adsorbent media to bind B2M and remove it from plasma [9]. These advances in technology have led to somewhat limited improvements in patient prognosis, with notable challenges in maintaining efficacy over extended durations of dialysis [10]. More recent studies have demonstrated the utility of using B2M binding proteins (including antibody fragments and nanobodies) or peptides attached to a matrix to sequester B2M and facilitate its removal [11,12,13,14,15]. Methods that remove B2M with greater specificity could improve efficacy without the unintended consequence of removing other similarly sized molecules in serum, including albumin and immunoglobulins.

We sought to investigate whether new ideas in protein engineering could be used to design a high-avidity soluble nanoparticle with favorable performance as an immunosorbent. To explore this, we investigated the viability of designed protein nanoparticles or nanocages, displaying B2M binding domains in a polyvalent fashion on their surface, for the removal of B2M from solution. We report on the successful creation of a novel protein nanoparticle for that purpose, along with important findings on stability and B2M binding and removal capabilities.

## 2. Methods

Cloning and Expression: DNA encoding B2M-adsorbent cages (BACs) was ordered from Twist Biosciences and cloned using Gibson assembly into the bacterial pET22b expression vector. Expression of nbBACs was performed by cloning sequence-verified BAC-encoding expression vectors into Shuffle T7 Express lysY cells (New England Biolabs) to allow for proper folding of disulfide bond-containing nanobodies. Cells were grown in autoinduction media [16] at 25 °C for 48 h. Cells were harvested at 4000× *g* and stored at −20 °C until purification.

BAC Purification: Frozen pelleted cells were solubilized in lysis buffer containing 50 mM TRIS pH 8.0, 250 mM NaCl, and protease inhibitor tablets (Thermo Fisher Scientific, Waltham, MA, USA). Cells were lysed using a C3 Emulsiflex (Avestin Inc., Ottowa, ON, CA) with four passages through the instrument. Lysate was clarified using centrifugation at 18,000× *g* for 35 min after which the supernatant fraction was passed over a gravity-flow column containing Ni-NTA functionalized agarose beads (Thermo Fisher Scientific, Waltham, MA, USA). The column was washed with lysis buffer containing imidazole of sequential concentrations: 50 mM, 75 mM and 100 mM. BACs were eluted from the column using lysis buffer supplemented with 500 mM imidazole. BAC proteins were then buffer-exchanged using dialysis into assay buffer containing: 50 mM TRIS, 150 mM NaCl and 0.02% Tween-20. SEC was performed to isolate correctly assembled nanoparticles using a Superose-6 column (Cytiva Life Sciences, Marlborough, MA, USA). The identity of fractions containing purified BACs were verified using SDS-PAGE. 

Purification of B2M: Avi and 6xHis-tagged B2M was expressed and purified using established methods with refolding [17,18]. B2M was then enzymatically biotinylated with the addition of BirA enzyme according to existing protocols.

Transmission Electron Microscopy (TEM): 4 μL of protein cage sample at 100 μg/mL were applied to glow-discharged Formvar/Carbon 300 mesh Cu grids (Ted Pella Inc., Redding, CA, USA) for one minute and then the excess liquid was blotted away. After a wash with 2% uranyl acetate, the grid was stained with 2% uranyl acetate for one minute. Images were taken on a Talos F200C (Thermo Fisher Scientific, Waltham, MA, USA). The same procedure was followed for the sample containing protein cage and B2M, in this case the final concentration of protein cage was 100 μg/mL and for B2M it was 50 μg/mL, giving a molar ratio of ~1:2 (protein cage:B2M).

Densitometry: Intensity of bands corresponding to B2M and BAC nanoparticle components co-eluting from SEC experiments were compared with intensities from lanes run containing known 1:1 stoichiometry of BAC binders to B2M protein.

Binding Affinity Measurements: Binding affinities were determined using an Octet RED96 BLI system (Sartorius AG, Göttingen, DE). Biosensors functionalized with streptavidin were used to bind biotinylated B2M at a concentration of 2.5 µg/mL and tested for binding with soluble BACs. Buffer used for experiments was 50 mM TRIS pH 8.0, 150 mM NaCl and 0.02% Tween-20.

Immunoblots: 3 µL aliquots of flowthrough samples from spin columns were applied to nitrocellulose paper, allowed to dry then repeated at the same location once. Established methods for Western blotting were then used to develop and image the blot. Anti-biotin antibodies were used to recognize biotinylated B2M (Invitrogen, Waltham, MA, USA). For Western blots, Mini-Protean Any-KD SDS-PAGE gels (Bio-Rad, Hercules, CA, USA) were used to separate proteins and then transferred to nitrocellulose membrane (Cytiva Life Sciences, Marlborough, MA, USA) using a Trans-Blot SD system (Bio-Rad, Hercules, CA, USA). The membrane was rocked in blocking buffer, i.e., TBST with 5% milk powder, for 1 h at room temperature with rocking. Primary anti-B2M antibodies were purchased from AbClonal (A1562) (Woburn, MA, USA) and used at a dilution of 1:1000 in blocking buffer. Membrane was allowed to incubate with primary antibody for 1 h at room temperature. The membrane was washed thrice in TBST, then incubated with anti-rabbit goat secondary antibody with HRP conjugate for an additional 1 h at room temperature. The membrane was washed of excess secondary antibody thrice with TBST, then incubated with Clarity Western ECL substrate (Bio-Rad, Hercules, CA, USA) and imaged on an Azure imager (Azure Biosystems, Dublin, CA, USA).

Size-excluded Flowthrough Experiment: 500 nM B2M with and without 1 µM BAC binders (i.e., 0.083 µM assembled BAC) was added to the supernatant of a centrifuge Amicon Ultra 100 kDa cutoff microcentrifuge concentrator, and then spun for 2.5 min at 4000× *g*. Flowthrough and supernatant were collected for further experimentation.

Serum Challenge Assay: SEC-purified BAC was added to human serum and incubated at 37 °C for 4 h. Serum mixture was then flowed over a column containing HisPur Ni-NTA resin (Thermo Fisher Scientific, Waltham, MA, USA) and washed and eluted with buffers identical to those used in IMAC purification. Elution fraction was analyzed using SDS-PAGE and concentrated and injected into a Superose-6 gel filtration column (Cytiva Life Sciences, Marlborough, MA, USA) for oligomeric state determination.

B2M Removal Experiment: 1 mL His-Trap columns (Cytiva Life Sciences, Marlborough, MA, USA) were used as a stationary matrix to immobilize BACs. Following incubation with SEC-purified BACs, human serum supplemented with 50 µg/mL of B2M was flowed over the column and the flowthrough was collected. Column was washed with binding buffer (50 mM Tris pH 8.0, 200 mM NaCl, 20 mM imidazole), and then eluted with elution buffer containing 50 mM Tris pH 8.0, 200 mM NaCl, 500 mM imidazole. These fractions were analyzed using Western blot according to the previously described protocol.

## 3. Results

### 3.1. Protein Design Approach

We first sought to generate novel protein nanoparticles displaying B2M binders on their surface. As a starting point for engineering, we focused on designed self-assembling nanoparticles often described in the literature as designed protein ‘cages’, owing to their open interiors, though their utility here derives from exterior attachment. To reflect their designed function, we refer to our constructs as B2M-adsorbent cages (BACs).

Designed protein nanocages have architectures that resemble viral capsids in certain respects (e.g., taking the forms of platonic solids such as a tetrahedron, cube or icosahedron) and have increasingly been at the heart of engineering studies that exploit their large size and symmetry [19,20,21,22,23,24,25,26]. Such cages are composed of tessellating protein molecules—typically one or two distinct protein subunit types each present in multiple copies—which self-assemble to form symmetric supramolecular assemblies or nanoparticles. Their structures make polyvalent attachment and display additional outward-facing proteins (or other molecule types), which is a relatively routine engineering process. We noted two potential benefits of applying protein cages to the problem of B2M removal technologies: (1) polyvalent display could lead to advantageous binding properties through well-known avidity effects (reviewed extensively by Varner, Kane and colleagues [27]), and (2) the large size of the nanocage could lead to unique opportunities for filtration, without removing beneficial plasma proteins (which are generally much smaller than the nanocage) in the process. There could also be advantages to technologies that bind and remove B2M while in solution rather than bound to highly dense stationary matrices, as the former approach might limit the chances of inadvertently creating and seeding B2M amyloid.

**Figure 1:**
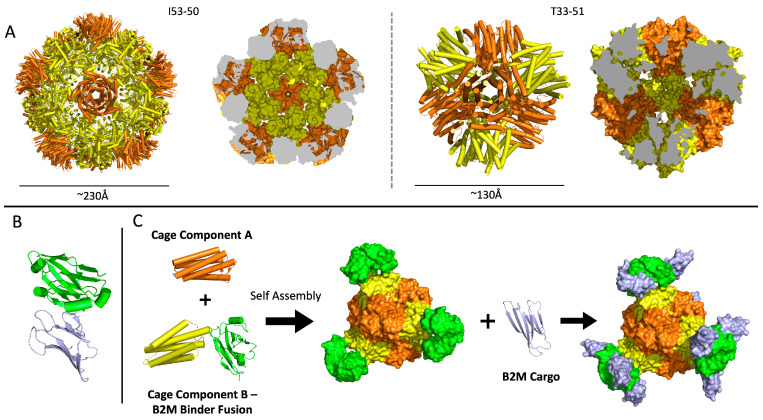
Design schema and building blocks of beta-2 microglobulin (B2M) adsorbent cage (BAC) nanoparticles. (**A**) Structural models of the I53-50 icosahedral protein cage and the T33-51 tetrahedral protein cage tested as scaffolds in several BAC designs. The protein structures are depicted as secondary structure cartoons (**left**) and as slices through space-filling models (**right**). (**B**) Structure of the anti-B2M nanobody (green) used in the design of BACs. B2M (purple) is shown in its bound configuration. (**C**) Design of a BAC from protein cage components (yellow and orange), with component B genetically fused to the anti-B2M nanobody. A fully assembled BAC shown in apo form (**middle**) as well as bound to B2M ligands (**right**).

For design and testing of BAC nanoparticles, we selected two previously characterized protein cages to serve as scaffolds for the development of functionalized nanoparticles. We generated initial designs based on two protein cages that have proven to be robust to various biochemical conditions and engineering modifications in our previous studies: the tetrahedral cage known as T33-51 [28] and the icosahedral cage known as I53-50 [29] (Figure 1A). The divergent sizes and symmetries of these candidates offered distinct engineering opportunities. Designs were generated by genetically fusing B2M binding nanobodies [30] to the outward-facing termini of the protein cage subunits, thereby creating nanobody BACs (nbBACs) (Figure 1B,C). Both of the candidate cages have a polypeptide chain terminus that extends to the outside surface of the cage, which was important to permit the scaffold to assemble correctly after modification, with the binding domains accessible on the exterior surface to interact with B2M. Note that both cages used here are of the two-component type, being comprised of two distinct subunit types A and B. Accordingly, the stoichiometry of the tetrahedral T33-51 cage is A12B12 while the icosahedral I53-50 cage has stoichiometry A60B60, meaning the former cage will display 12 copies of a binding domain on its surface whereas the latter cage will display 60 copies.

For the first round of nbBACs, we employed a minimal polypeptide linker (a GGS sequence) to connect a C-terminus of a protein cage component to the N-terminus of the B2M nanobody. For these starting candidates, we refer to the nanoparticle based on the smaller T33-51 cage as nbBAC1_SL (for short linker) and the nanoparticle based on the I53-50 cage as nbBAC2 (Supplemental Data Table S1).

### 3.2. Testing and Design Optimization on Cages

**Figure 2:**
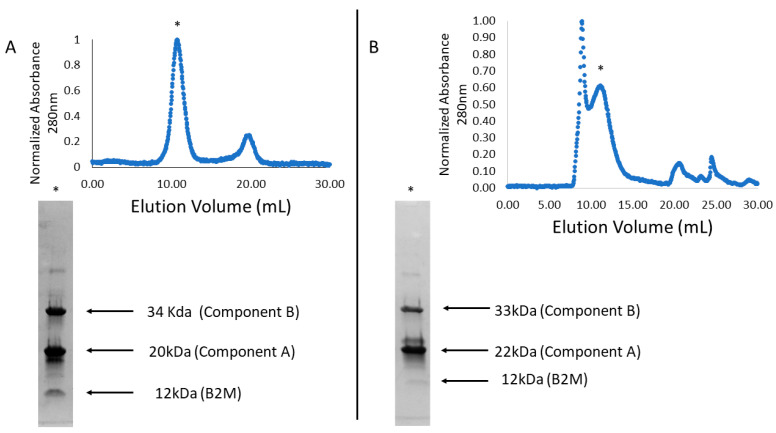
Assembled BAC nanoparticles bind to and co-elute with B2M cargo. (**A**) nbBAC1_SL mixed with B2M elutes from Superdex 200 SEC column (Cytiva Life Sciences) at a volume consistent with proper cage assembly (denoted with asterisk). SDS-PAGE analysis of the cage peak fraction indicates B2M co-elutes with BAC. (**B**) nbBAC2 mixed with B2M elutes from Superose-6 SEC column (Cytiva Life Sciences) at a volume consistent with proper cage assembly (denoted with asterisk). SDS-PAGE analysis of the cage peak fraction indicates B2M co-elutes with BAC.

The proteins designed and analyzed here were over-expressed in *Escherichia coli* cells and purified using affinity chromatography (see Methods). Note that our designed BACs are intended to self-assemble and to therefore appear in their assembled forms upon expression and purification. Size-exclusion chromatography (SEC) was used to assay assembly, comparing elution volumes of peaks containing BAC components to known elution volumes for the original unmodified cages used as scaffolds. For binding tests, our BACs were mixed with purified B2M protein and assessed for binding using SEC. In the event of a stable interaction between the BAC and B2M, co-elution of proteins would be expected. If no interaction occurs, B2M would be expected to elute much later due to its significantly smaller size relative to the BAC assembly. For experiments on both of our initial BAC designs (nbBAC1_SL and nbBAC2), we noted visible co-elution of B2M with the proteins making up each of the BAC assemblies (Figure 2). However, closer examinations led to two important observations on the first round of nbBAC designs: (1) nbBAC1_SL displayed limited stability in its assembled state, precipitating out of solution over time, and exhibited a propensity to rapidly disassemble into smaller subunits (Supplemental Data Figure S1); and (2) nbBAC2 displayed sub-stoichiometric binding to B2M when visualized on SDS-PAGE. For purifications of nbBAC2 analyzed using SEC, a large void peak, indicative of aggregation and partially overlapping with the BAC peak was also present (Figure 2B). These observations, which would have limited downstream utility, motivated a second round of designs.

**Figure 3:**
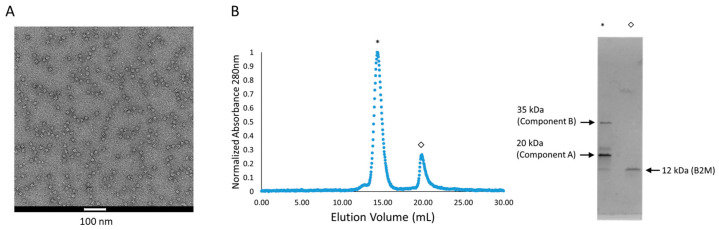
Characterization, purification and B2M binding of an improved B2M-binding nanoparticle, nbBAC1_LL. (**A**) Negatively stained TEM image of assembled SEC-purified nbBAC1_LL nanoparticles. (**B**) The improved nbBAC1_LL, mixed with B2M, elutes in SEC (**left**) at a volume consistent with proper cage assembly (denoted by an asterisk). SDS-PAGE analysis (**right**) of the cage peak fraction indicates B2M co-elutes with BAC.

We rationalized that the issues with nbBAC1_SL stability may have been the result of steric clashes between neighboring nanobody domains projecting from the assembled cage via short linkers. To test that hypothesis, we generated a new BAC design based on nbBAC1, but with a longer 12-residue (GS)6 linker, which we designated as nbBAC1_LL (long linker) (Supplemental Data Table S1). We went on to test the assembly and binding properties of nbBAC1_LL using negatively stained transmission electron microscopy (TEM) and size exclusion chromatography (SEC) and noted improved stability of this BAC as well as a higher yield of protein eluting at the expected elution volume for a tetrahedral cage assembly (Figure 3A, Supplemental Data Figure S1). We confirmed assembly was not altered by B2M binding using TEM and SEC on nbbBAC1_LL mixed with B2M (Figure 3B, Supplemental Data Figure S2). Densitometric analysis of SDS-PAGE gels from B2M co-eluting with assembled BAC on SEC compared with gels of known 1:1 stoichiometry revealed an approximate ratio of 2:1 stoichiometry of B2M binders to B2M protein. In other words, mixing excess B2M with BAC proteins resulted in roughly half occupancy of our BAC binders, corresponding to about six B2M molecules bound to an individual nanoparticle. This improved nanoparticle was evaluated more extensively in further experiments (discussed subsequently).

The larger BAC design, nbBAC2 based on the I53-50 cage, suffered from a limited capacity for B2M binding. In this case, we hypothesized that the binding site for the nanobody may have been partially occluded by the scaffold, so we made use of the presence of both N- and C-termini on the outside of the I53-50 protein cage scaffold to generate an alternative nbBAC where the nanobody was fused through its N-terminus rather than the C-terminal fusion used for nbBAC1 and nbBAC2, thus generating nbBAC2N (Supplemental Data Table S1). However, this alternative construct displayed limited expression and solubility and no visible assembly into icosahedral geometry. Considering its apparent inability to support the fusion of exterior nanobody domains, the I53-50 cage was abandoned as a basis for our BACs.

### 3.3. Assays on nbBAC1_LL for B2M Removal

**Figure 4:**
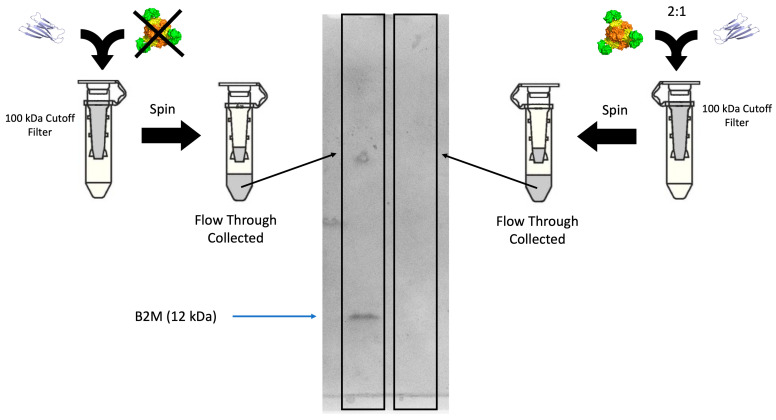
Binding of B2M with nbBAC1_LL in a B2M retention assay. nbBAC1_LL binds to soluble B2M and retains it in the supernatant of a concentrator column with a molecular weight cutoff of 100 kDa (**right**). When no nbBAC is added to the column, B2M flows through the membrane unimpeded (**left**). B2M has a MW of 13.8 kDa, and nbBAC has a MW of 662 kDa. An SDS-PAGE gel shows the composition of the flowthrough in each experiment. B2M is only present in the gel run on the flowthrough of the experiment without nbBAC added, showing that B2M is fully retarded by the interaction between nbBAC1_LL and B2M.

To characterize the ability of our leading BAC candidate, nbBAC1_LL, to bind and sequester B2M, we performed a size-based retention assay utilizing a centrifugal filter with a molecular weight cutoff of 100 kDa, which falls between the size of B2M and our BAC designs. When centrifuged, a stable interaction between B2M and BAC should prevent B2M from passing through the filter, whereas B2M alone should pass through the filter unimpeded (Figure 4). That is, adding the BAC to a solution of B2M should prevent any protein material from passing through the size filter. Our experiments confirmed that behavior. Using a BSA assay, we measured the protein concentration in the flowthrough of both experiments, testing 500 nM B2M plus or minus addition of nbBAC1_LL at 1 µM. The resulting protein concentration in the flowthrough was determined to be at least 5 times lower when nbBAC1_LL was added (above the centrifugal filter) compared to when it was absent. The BAC effectively sequesters the B2M in a large complex that is unable to pass through the filter membrane. Owing to the detection limit of the BSA assay, the precise depletion factor for removal of B2M in this size-based experiment is likely considerably higher than the limiting value of five noted above. Indeed, B2M was undetectable using immunoblot in the flowthrough fraction; chemiluminescent signal is only visible on the blot containing flowthrough from the experiment without nbBAC1_LL added to the B2M (Supplemental Data Figure S3).

We investigated the binding of B2M to nbBAC1_LL using biolayer interferometry (BLI). In order to avoid potential artifacts—e.g., cage disruption and/or occlusion of binding sites—that might occur by attaching the BAC to the BLI sensor tip, we elected instead to attach B2M to the tip and monitor the binding of nbBAC1_LL following standard protocols (see Methods). We obtained binding kinetic curves under a series of nbBAC1_LL concentrations, ranging from 1 nM to 1000 nM. For concentrations in the range of 1 nM to 100 nM, we obtained excellent fits to theoretical curves for binding to identical and independent binding sites (Supplemental Data Figure S4). In contrast, for concentrations of mbBAC1_LL higher than 100 nM, we obtained binding curves inconsistent with simple binding phenomena. In particular, attempts to fit response curves to a model based on identical and independent binding sites illuminated a tighter binding for occupation of initial sites and weaker binding for occupation of subsequent sites. We interpreted this negative cooperative effect to steric occlusion in binding multiple BACs to the sensor tip; the ligand (nbBAC1_LL in this case) is much larger than in typical BLI experiments. From the data at moderate concentrations of nbBAC1_LL, the binding affinity (Kd) was measured to be 4.2 nM (+/−0.3 nM). This presents an improvement by about an order of magnitude over the published Kd value of 58 nM for the same nanobody when used in isolation [30]. The complexity of our polyvalent system similarly complicated our interpretation of kinetic behavior in terms of individual molecular events. Notwithstanding this, in the regime of moderate concentrations of the BAC, a kinetic off rate of 1.85 × 10^−4^ s^−1^ ± 3 × 10^−6^ s^−1^ was obtained. We further assessed whether this designed BAC would remain stable under conditions relevant for dialysis applications. We incubated nbBAC1_LL at 37 °C in human serum for 4 h and then assayed its oligomeric state. After pulling down nbBAC1_LL based on its polyhistidine tag using Ni-NTA resin, and then eluting with imidazole, we analyzed the major species by SEC and established that the size corresponded to that of the natively assembled BAC nanoparticle (Supplemental Data Figure S5). The designed nbBAC1_LL nanoparticle therefore appears stable to those conditions.

**Figure 5:**
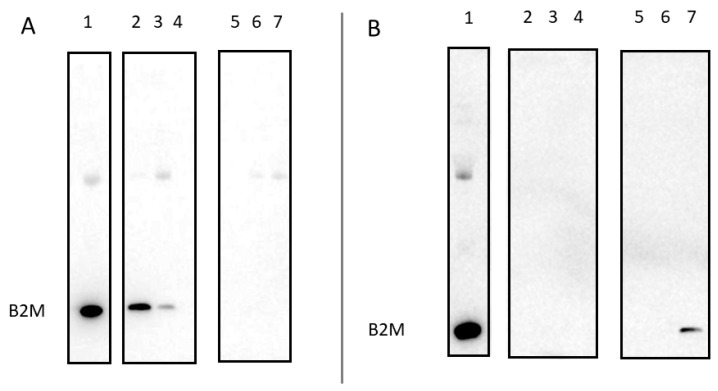
Nanoparticle nbBAC1_LL bound to a stationary matrix fully removes B2M from human serum. SDS-PAGE gels are analyzed using Western blotting with an anti-B2M antibody. (**A**) Analysis of sequential flowthrough fractions of serum supplemented with B2M flowed over resin, either with nbBAC1_LL bound to the column (lanes 5–7) or without (lanes 2–4). Lane 1 is 20× diluted serum supplemented with B2M. (**B**) Analysis of resin-bound components from serum supplemented with B2M, eluted from the resin using 1M imidazole buffer, either with nbBAC1_LL bound to the column (lanes 5–7) or without (lanes 2–4).

In clinical dialysis applications, the slow speed of filtering proteins by size-selective membranes can be limiting, motivating applications based on selective removal of proteins flowing over a stationary binding matrix. Accordingly, to evaluate its capacity as a component of a binding matrix, we immobilized nbBAC1_LL on a metal affinity (His-trap) column and then flowed human serum supplemented with 50 µg/mL B2M over the matrix and tested the flowthrough for B2M. We also tested the column eluate after washing off the BAC from the column using imidazole. We analyzed samples for B2M using Western blotting and observed B2M only in the BAC elution fraction, with no detectable amounts in the flowthrough (Figure 5). This shows that, under the conditions tested, which were intended to mimic the serum of afflicted dialysis patients, B2M is practically completely removed by a single passage over the nbBAC1_LL-bound material.

## 4. Discussion

A key aim of the present study was to design and evaluate a new type of protein-based polyvalent binding material that might be suitable for depleting human plasma of certain proteins, particularly B2M, which accumulates to toxic levels and then forms pathogenic amyloid deposits in long-term dialysis patients. We chose designed protein cages as a framework for creating such materials, to exploit their size and their polyvalent avidity when modified with binding domains. Among protein cages that have been created to date, a few have proven amenable to further modification to display fused proteins on their outer surfaces [25,27,31,32,33]. That is, they have outwardly disposed chain termini and retained their stability and self-assembling properties after fusion of additional protein domains; many protein cages do not meet those criteria. In the present study, we found it necessary to examine multiple candidate protein cages and fusion strategies (e.g., linker lengths) to obtain a robust nanoparticle with favorable B2M binding properties. The specific construct demonstrated here may be a useful starting point for clinical testing.

The best performing nanoparticle, nbBAC1_LL, complexes with B2M to produce an approximately 732 kDa assembly with a demonstrated binding capacity of about six copies of B2M on its exterior. We showed that the nanoparticle has the ability to remove B2M in a size-based separation, i.e., by employing a semi-permeable membrane filter that would allow smaller proteins (e.g., albumin) to pass. For possible flow-based applications, we envisage that this nanoparticle could be affixed to a stationary matrix. Note that in the experiments here we affixed our BAC to a nickelated matrix based on polyhistidine tails; for clinical applications, a more specific attachment might be important. A straightforward option would be to attach a biotinylated version of our BAC to a polymeric matrix functionalized with streptavidin. Streptavidin has been employed extensively as a platform for chemical biology and medical device applications [34]. Such a medium could allow blood to pass through but would retain B2M bound to the stationary phase. This could allow it to be used in series with existing dialyzers with relatively minor modification of existing techniques. Like other immunosorbents, the column could be reused by eluting BAC nanoparticles, followed by sterilization and regeneration with fresh BAC nanoparticles. The experiments in the present study emphasize the effectiveness of our BACs in removing B2M potentially with only one passage of serum over the medium. It is notable that most existing size-based filtration methods involve passage of blood over filtration membranes up to 20 times, while realizing considerably less reduction in B2M concentrations. The materials described here might therefore offer advantages for lowering B2M levels and helping to prevent DRA amyloidosis in patients on chronic dialysis.

Regarding limitations, as demonstrated so far, our BAC materials are only semi-renewable. Whereas a stationary matrix should ideally be fully regenerative, we have thus far not demonstrated a method to dissociate B2M, tightly bound to BACs, without sacrificing the integrity of the BAC nanoparticles. Such an improvement could be critical, given the large quantities of B2M (on the range of hundreds of milligrams) present in one blood volume of an afflicted patient. In summary, with suitable modifications to address issues of regeneration, the novel nanoparticle platform described here could find utility, in conjunction with existing dialysis protocols, in alleviating DRA amyloidosis in large populations subject to long-term dialysis.

## 5. Patents

TOY, JEM, RCG, MAA, AM, BN and PM anticipate a patent filing by UCLA on the materials described.

## Data Availability

The data presented in this study are available within article and Supplementary Material.

## Supplementary Materials

The following supporting information can be downloaded at: https://www.mdpi.com/article/10.3390/biom13071122/s1, Figure S1: SEC elution profiles of BAC nanoparticles; Figure S2: Negatively stained TEM image of nbBAC1_LL mixed with B2M cargo at a ratio of 2:1 (B2M:cage); Figure S3: Immunoblot analysis of flowthrough from the B2M retention assay; Figure S4: Measurement of binding affinity of B2M to nbBAC1_LL; Figure S5: nbBAC1_LL is stable in human serum. Table S1: BAC sequences.
