# Supplementary material for: Design of Beta-2 Microglobulin Adsorbent Protein Nanoparticles
Source: Biomolecules. 2023 Jul 14;13(7):1122. doi: 10.3390/biom13071122 (PMC10377675; doi:10.3390/biom13071122)
Supplement: Supplementary file 1 [file biomolecules-13-01122-s001.zip › biomolecules-2461420-supplementary.pdf]

Supplemental Materials for

Design of Beta-2 Microglobulin Adsorbent Protein Cages

Justin E. Miller, et al.

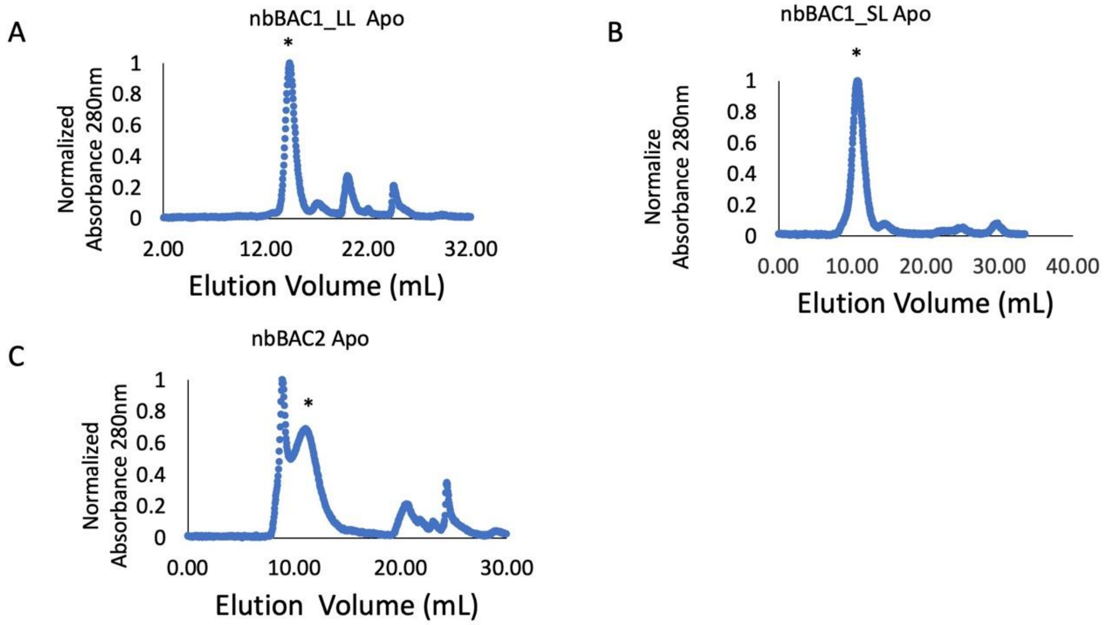

Supplemental Data Figure 1. SEC elution profiles of BAC nanoparticles. SEC chromatograms for nbBAC1_LL (A), nbBAC1_SL (B), and nbBAC2_SL (C). Assembled BAC nanoparticles are denoted with asterisks.

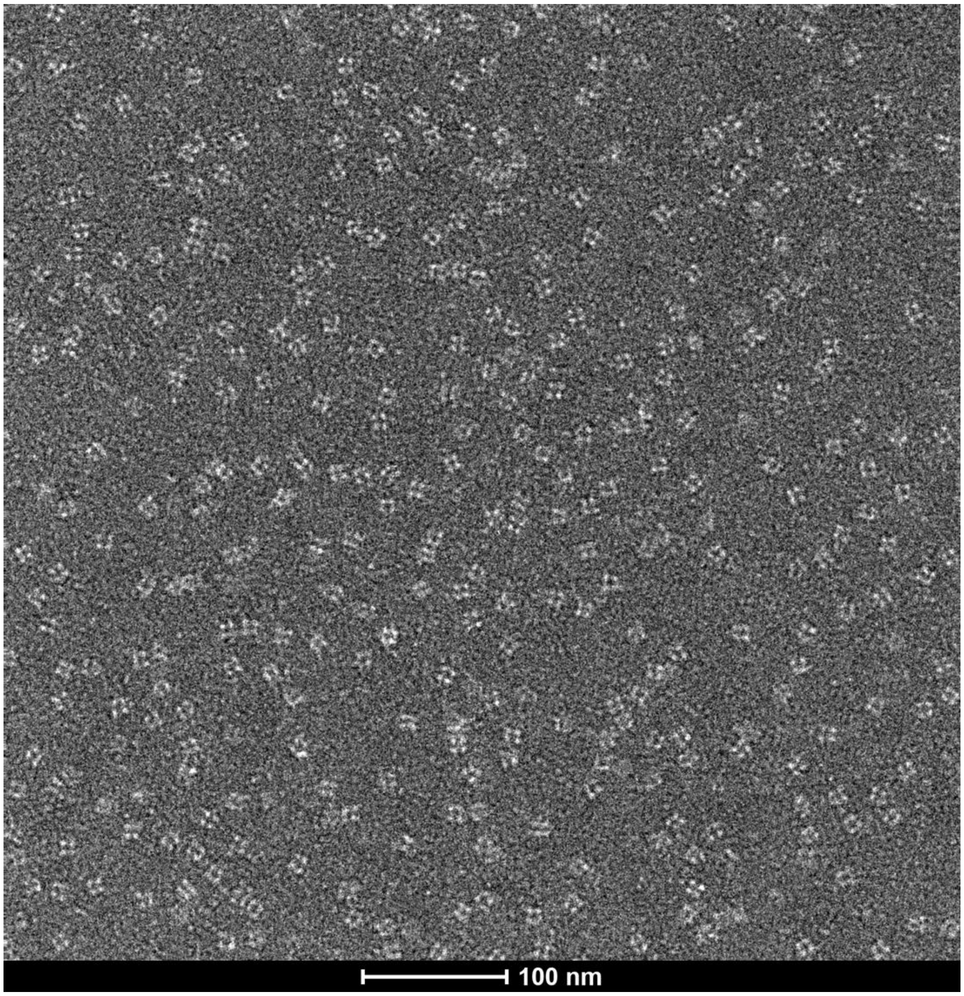

Supplemental Data Figure 2. Negatively stained TEM image of nbBAC1_LL mixed with B2M cargo at a ratio of 2:1 (B2M:cage).

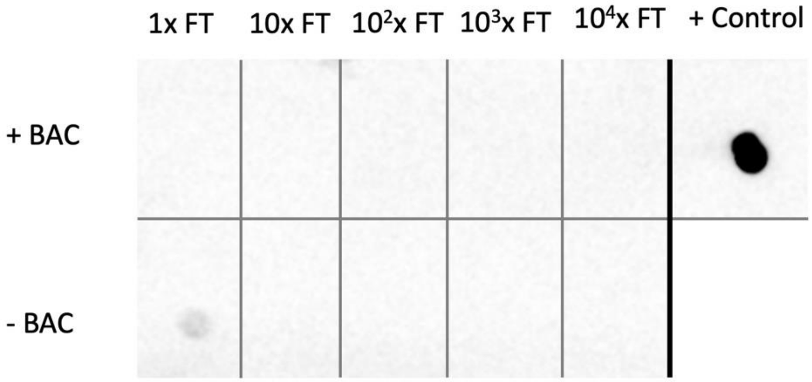

Supplemental Data Figure 3. Immunoblot analysis of flowthrough from the B2M retention assay. Serial dilutions of flow through from the size-filtration retention assay either with (top) or without (bottom) nbBAC1_LL added to supernatant were analyzed for B2M via immunoblot. A positive control (1uM B2M) is depicted in top right.

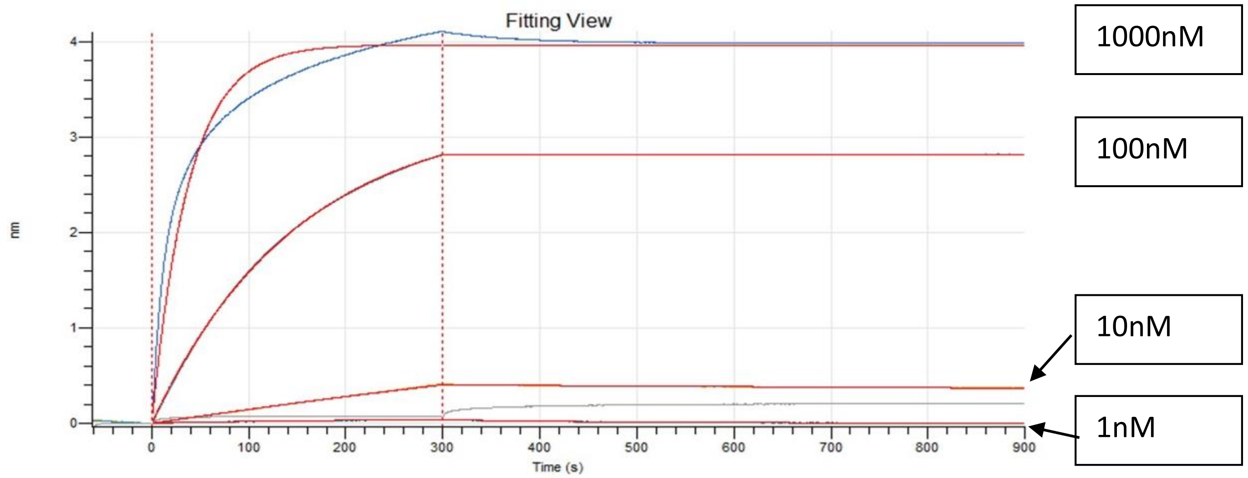

Supplemental Data Figure 4. Measurement of binding affinity of B2M to nbBAC1_LL. Biolayer interferometry was used to estimate the equilibrium dissociation constant of nbBAC for B2M. Based on experiments with BAC concentrations between 1 nm and 100 nM, the estimated Kd value is 4.2 nM (+/- 8 nM). Curves corresponding to BAC concentrations of 1000nM, 100nM, 10nM, and 1nM are shown.

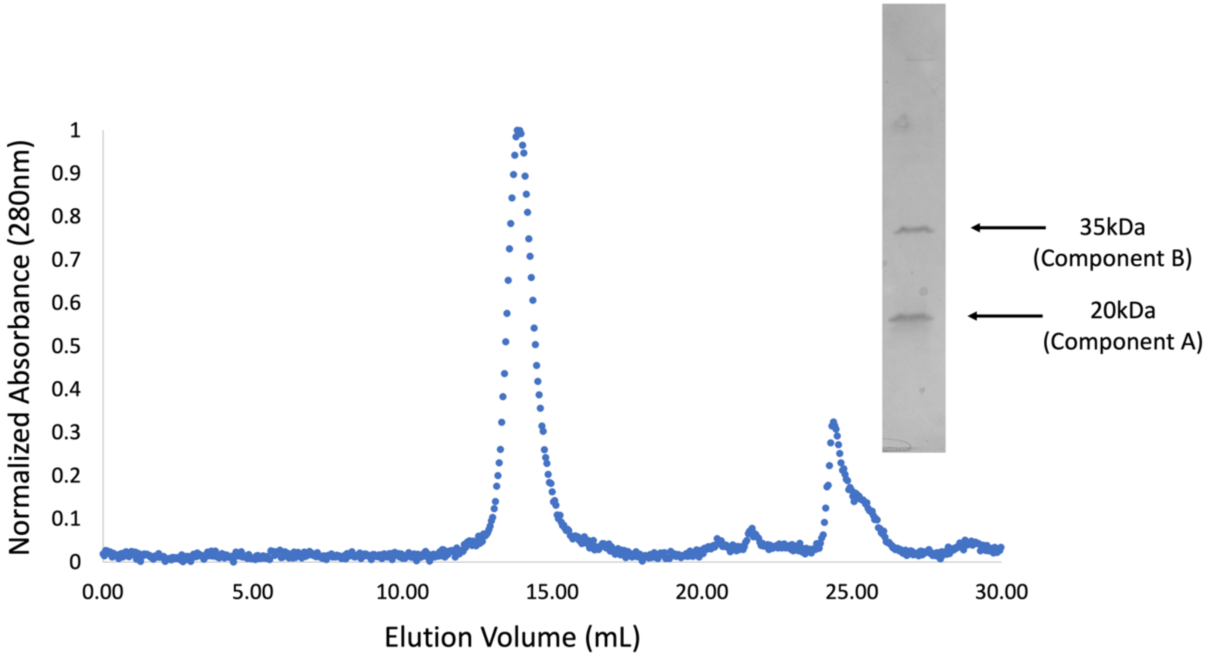

Supplemental Data figure 5. nbBAC1_LL is stable in human serum. nbBAC1_LL incubated in serum is purified and analyzed by SDS-PAGE and by SEC using Superose-6 column indicating nbBAC1_LL maintains correct assembly geometry in dialysis conditions.

**nbBAC1_SL**	>A component<br>MFTRRGDQGETDLANRARVGKDSPVVEVQGTIDELNSFIGYALVLSR<br>WDDIRNDLFRIQNDLFVLGEDVSTGGKGRTVTMDMIIYLIKRSVEMKA<br>EIGKIELFVVPGGSVESASLHMARAVSRRLERRIKAASELTEINANVLL<br>YANMLSNILFMHALISNKRLNIPEKIWSIHRVSLE<br>>B component<br>MRITTKVGDKGSTRLFGGEEVWKDDPIIEANGTLDELTSFIGEAKHYV<br>DEEMKGILEEIQNDIYKIMGEIGSKGKIEGISEERIKWLAGLIERYSEMV<br>NKLSFVLPGGTLESAKLDVCRTIARRAERKVATVLREFGIGTLAAIYLA<br>LLSRLLFLLARVIEIEKNKLKEVRSGGSQVQLQESGGGSVQAGGSLRL<br>SCAASGYTDSRYCMAWFRQAPGKEREWVARINSGRDITYYADSVKG<br>RFTFSQDNAKNTVYLQMDSLEPEDTATYYCATDIPLRCRDIVAKGGD<br>GFRYWGQGTQVTVSSHHHHHH
**nbBAC1_LL**	>A component<br>MFTRRGDQGETDLANRARVGKDSPVVEVQGTIDELNSFIGYALVLSR<br>WDDIRNDLFRIQNDLFVLGEDVSTGGKGRTVTMDMIIYLIKRSVEMKA<br>EIGKIELFVVPGGSVESASLHMARAVSRRLERRIKAASELTEINANVLL<br>YANMLSNILFMHALISNKRLNIPEKIWSIHRVSLE<br>>B component<br>MRITTKVGDKGSTRLFGGEEVWKDDPIIEANGTLDELTSFIGEAKHYV<br>DEEMKGILEEIQNDIYKIMGEIGSKGKIEGISEERIKWLAGLIERYSEMV<br>NKLSFVLPGGTLESAKLDVCRTIARRAERKVATVLREFGIGTLAAIYLA<br>LLSRLLFLLARVIEIEKNKLKEVRSGGSGGGSGGGGSQVQLQESGGG<br>SVQAGGSLRLSCAASGYTDSRYCMAWFRQAPGKEREWVARINSGR<br>DITYYADSVKGRFTFSQDNAKNTVYLQMDSLEPEDTATYYCATDIPLR<br>CRDIVAKGGDGFRYWGQGTQVTVSSHHHHHH
**nbBAC2**	>A component<br>MKMEELFKKHKIVAVLRANSVEEAIEKAVAVFAGGVHLIEITFTVPDAD<br>TVIKALSVLKEKGAIIGAGTVTSVEQCRKAVESGAEFIVSPHLDEEISQF<br>CKEKGVFYMPGVMTPTELVKAMKLGHDILKLFPGEVVGPQFVKAMK<br>GPFPNVKFVPTGGVNLDNVCKWFKAGVLAVGVGKALVKGKPDEVRE<br>KAKKFVKKIRGCTE<br>>B component<br>MNQHSHKDHETVRIAVVRARWHAEIVDACVSAFEAAMRDIGGDRFA<br>VDVFDVPGAYEIPLHARTLAETGRYGAVLGTAFVVNGGIYRHEFVASA<br>VINGMMNVQLNTGVPVLSAVLTPHNYDKSKAHTLLFLALFAVKGMEA<br>ARACVEILAAREKIAAGSGGSGGSGGSQVQLQESGGGSVQAGGSLR<br>LSCAASGYTDSRYCMAWFRQAPGKEREWVARINSGRDITYYADSVK

	GRFTFSQDNAKNTVYLQMDSLEPEDTATYYCATDIPLRCRDIVAKGG<br>DGFRYWGQGTQVTVSSAHHSEDPHHHHHH
**nbBAC2N**	>A component<br>MKMEELFKKHKIVAVLRANSVEEAIEKAVAVFAGGVHLIEITFTVPDAD<br>TVIKALSVLKEKGAIIGAGTVTSVEQCRKAVESGAEFIVSPHLDEEISQF<br>CKEKGVFYMPGVMTPTELVKAMKLGHDILKLFPGEVVGPQFVKAMK<br>GPFPNVKFVPTGGVNLDNVCKWFKAGVLAVGVGKALVKGKPDEVRE<br>KAKKFVKKIRGCTE<br>>B component<br>MQVQLQESGGGSVQAGGSLRLSCAASGYTDSRYCMAWFRQAPGK<br>EREWVARINSGRDITYYADSVKGRFTFSQDNAKNTVYLQMDSLEPED<br>TATYYCATDIPLRCRDIVAKGGDGFRYWGQGTQVTVSSGGSGGSGG<br>SNQHSHKDHETVRIAVVRARWHAEIVDACVSAFEAAMRDIGGDRFAV<br>DVFDVPGAYEIPLHARTLAETGRYGAVLGTAFVVNGGIYRHEFVASAV<br>INGMMNVQLNTGVPVLSAVLTPHNYDKSKAHTLLFLALFAVKGMEAA<br>RACVEILAAREKIAAGSHHHHHH

Supplemental Data Table 1 BAC sequences.
